# Regenerative Endodontics and Minimally Invasive Dentistry: Intertwining Paths Crossing Over Into Clinical Translation

**DOI:** 10.3389/fbioe.2022.837639

**Published:** 2022-02-08

**Authors:** Hisham Elnawam, Menatallah Abdelmougod, Ahmed Mobarak, Mai Hussein, Hamdy Aboualmakarem, Michael Girgis, Rania El Backly

**Affiliations:** ^1^ Endodontics, Conservative Dentistry Department, Faculty of Dentistry, Alexandria University, Alexandria, Egypt; ^2^ Tissue Engineering Laboratories, Faculty of Dentistry, Alexandria University, Alexandria, Egypt

**Keywords:** minimally invasive endodontics, regenerative endodontic procedures, biomimetic scaffolds, biomimetic disinfection protocols, stem cells, immature necrotic permanent teeth, tooth structure preservation

## Abstract

Regenerative endodontic procedures have been described for over a decade as a paradigm shift in the treatment of immature necrotic permanent teeth, owing to their ability to allow root maturation with subsequent enhancement of the tooth’s fracture resistance in addition to the potential for regeneration of vital intracanal tissues. Concomitantly, minimally invasive endodontics is another rising concept with the main concern of preservation of tooth structure. Stemming from their potential to preserve the original tooth structure, both regenerative and minimally invasive endodontics could be considered as two revolutionary sciences with one common goal. Achieving this goal would entail not only employing the appropriate strategies to recreate the ideal regenerative niche but modifying existing concepts and protocols currently being implemented in regenerative endodontics to address two important challenges affecting the outcome of these procedures; conservation of tooth structure and achieving effective disinfection. Therefore, the search for new biomimetic cell-friendly disinfecting agents and strategies is crucial if such a novel integratory concept is to be foreseen in the future. This could be attainable by advocating a new merged concept of “minimally invasive regenerative endodontic procedures (MIREPs),” through modifying the clinical protocol of REPs by incorporating a minimally invasive access cavity design/preparation and biomimetic disinfection protocol, which could enhance clinical treatment outcomes and in the future; allow for personalized disinfection/regeneration protocols to further optimize the outcomes of MIREPs. In this review, we aim to introduce this new concept, its realization and challenges along with future perspectives for clinical implementation.

## Introduction

### Regenerative Endodontics and Minimally Invasive Dentistry in the Wake of the COVID-19 Pandemic

At the end of the year 2019, the Severe Acute Respiratory Syndrome Coronavirus-2 (SARS-COV-2) virus changed the face of humanity (Ather et al., 2020). Interestingly, a newly defined role for regenerative therapies has re-emerged with the potential of tissue engineering and regenerative medicine to play a critical role in improving clinical practices amid a global viral outbreak. This can range from the development of *in-vitro* models for drug testing and disease modeling, designing drug delivery systems to optimizing vaccine delivery platforms (Tatara, 2020).

In particular, the dental community faced unique challenges, leading to the development of treatment strategies changes to reduce the risks of virus transmission to patients/dental practitioners (Bahador et al., 2021). In endodontics, a shift towards minimally invasive endodontic procedures was suggested over more invasive modalities highlighting a new role for these therapies in light of the COVID-19 pandemic (Azim et al., 2020).

### The Dental Pulp: A Unique Commodity

While the dental pulp is a unique resilient tissue responsible for the survival of one of the human body’s most important organs; the tooth, it is often undervalued in routine dental practice. While vital pulp therapies have been routinely implemented in daily endodontic practice for years, performing a partial/complete pulpotomy for mature irreversibly inflamed permanent teeth has been a procedure fraught with scepticism over the past years. Recent evidence highlights that these treatments may present a paradigm shift in endodontic therapies in the upcoming era (Duncan et al., 2019a). Congruently, REPs which have been employed as a predictable treatment for immature necrotic teeth for years, have slowly seen applications for mature necrotic permanent teeth as well (El-Kateb et al., 2020; Glynis et al., 2021). Indeed, the past 2 years have witnessed an incredible increase in publications about REPs citing procedures supporting single visit procedures over multiple visit ones (Cerqueira-Neto et al., 2021).

Although all REPs have been practiced for some time, seasoned endodontists and dental practitioners were always reluctant to apply these techniques in their routine dental practices, dreading the lack of predictability and due to perceptions, on shortcomings of available materials and restorative options. With the advent of advanced tricalcium-silicate cements and sophisticated bonding and restorative materials, successful long-term outcomes are being increasingly documented with reproducibility and obvious advantages for both the patient and the practitioner. This new direction is currently on its way to embedding firm roots in the field with a mental makeshift from a science of “maybe” to a science that has actually transitioned into routine clinical practice; ironically pushed forward by a global pandemic. In this review, we introduce a new concept amalgamating the sciences of MIEs with REPs in the hope of paving the way to strategies that will not only regenerate lost tooth structures but conserve those that still remain as well.

## Regenerative and Minimally Invasive Endodontic Procedures: Two Revolutionary Sciences With One Common Goal

### Minimally Invasive Endodontics: From Tooth Preservation to Tooth Survival

Minimally invasive endodontics (MIEs) concept is based on the assumption that tooth structure preservation during access cavity preparation/canal instrumentation is a vital parameter to maintain strength and fracture resistance of the tooth. Contracted endodontic cavity (CEC) designs conserve a larger amount of the coronal tooth structure, thus reducing stress concentration on the occlusal and cervical areas of the tooth (Yuan et al., 2016). Moreover, minimal canal preparation with less taper contributes to the preservation of more cervical dentin which in turn could enhance the resistance of the tooth to fracture under masticatory loads (Yuan et al., 2016; Silva et al., 2020a). Previous finite element analysis studies performed on standardized virtual models showed larger stress concentration areas in the cervical region of teeth with traditional endodontic cavity (TEC) compared to CEC (Silva et al., 2020a). Despite the benefits, there are controversies in literature concerning the cleaning effectiveness and difficult exploration of root canal systems through these accesses, affecting the longevity of teeth from a biological point of view (Silva et al., 2020a).

### Regenerative Endodontics as a Minimally Invasive Treatment Modality

Regenerative endodontic procedures (REPs) utilize the concept of tissue engineering to restore the root canal system to a healthy state, allowing for the continued development and regeneration of the root and surrounding tissues (He et al., 2017). Indeed, the primary objectives of REPs mainly address the conservation and preservation of the remaining tooth structure as well as enhancing its survival, targeting the resolution of Apical Periodontitis (AP), induction of apical closure and increased root canal wall thickness and length of immature teeth (Endodontistso AAo, 2016). Regarding these objectives, REPs and MIEs endodontic concepts relatively share an equivalent aim of conservation and preservation of teeth in a functional state. In other words, REPs aim to restore the original tooth structure while MIEs aim to preserve it.

A recent suggestion has been to redefine the definition of regenerative endodontics as follows: “The term regenerative endodontics should embrace the repair, replacement and regeneration of dentin–pulp lost due to age, disease, trauma or congenital defects to restore normal function” (Duncan et al., 2019b).

Theoretically, if we can sterilize the canal, and recreate a 3D “biological filling” in the form of regenerated vital tissues this would ensure an optimum outcome. Regenerative strategies targeting mature teeth will most likely differ from those for immature permanent teeth, due to the different challenges encountered in both situations regarding the weak natural architecture of immature teeth, in addition to, the complexity of biofilms, toxins, and antigens which could reside in the larger dentinal tubules in these teeth (He et al., 2017; Verma et al., 2017; Almutairi et al., 2019; Fouad, 2020). Excessive removal of tooth structure accompanying a TEC especially at pulp chamber walls and around the canal orifices, may decrease the tooth resistance to fracture during function. This fact could be more prominent in REPs where the bulk of dentin is initially much less compared to mature teeth (Elkholy et al., 2021).

Furthermore, the preservation of peri-cervical dentin in particular which has an anatomical pattern of a lesser inter-tubular dentin and wider dentinal tubule lumen can justify the evidence of greater sequestered growth factors including TGF-β1, FGF2, and VEGF found in this region (Tziafas et al., 2019; Ferreira et al., 2020). This would thus preserve the physiologic and defensive function of dentin which has been proven to have a defensive mechanism that involves the release of pro-inflammatory cytokines and antibacterial agents (Ayoub et al., 2020; Shah et al., 2020). This coupled with the ability to perform REPs in canals with apical diameters as narrow as 0.3 mm offers the possibility to apply a combined regenerative and minimally invasive clinical protocol without the fear of leaving behind residual infected tissues (El-Kateb et al., 2020).

Therefore, combining MIEs and REPs might be considered a logical and new idea. On the other hand, it may complicate the outcomes of REPs due to the limitation of the cleaning and disinfection of the root canal system which could be encountered through the CEC. Hence, if such a strategy is to be employed, considerations must not only be given to how modification of the access cavity will influence adequate disinfection, but also should encourage single visit treatment modalities via the application of biologically inspired and biomimetic scaffolds to guarantee tissue regeneration in a bacteria-free milieu.

## Disinfection and Regeneration; a Cause-and-Effect Relationship

### How Detrimental Really is Residual Bacterial Biofilm?

The vast majority of failed regenerative endodontic treatment cases have been diagnosed with necrotic pulp with/without some form of apical pathosis which strongly suggests the association between persistent root canal infection and treatment failure (Almutairi et al., 2019). Concomitantly, complete root canal disinfection in regenerative endodontics in immature teeth is quite challenging as mechanical debridement entirely relies on the use of minimal mechanical instrumentation to avoid further weakening of the root. Therefore, it is possible that bacteria are incompletely eradicated. Yet, unlike conventional root canal treatment (RCT), lowering intracanal bacterial threshold might not be sufficient for the success of REPs as the residual bacteria might repopulate leading to treatment failure (Verma et al., 2017; Ruparel et al., 2019). Another barrier to regeneration is the effect of residual infection on the ideal fate of stem cells (Verma et al., 2017; Lui et al., 2020). Bacteria and their antigens could modify stem cell differentiation into osteoblastic phenotype and hinder their mineralizing capacity (Vishwanat et al., 2017). Additionally, remaining pathogens alter the induced release of TGF-β1 from dentin (Cameron et al., 2019). Bacterial lipopolysaccharides could also remain after root canal disinfection and promote pro-inflammatory cytokines production (Kato et al., 2014). Additionally, induction of an inflammatory periapical plug was demonstrated to hinder cell migration into the root canal and subsequently prevent tissue regeneration (Zaky et al., 2020). Hence, a prerequisite for dentin-pulp tissue regeneration is creating a pathogen-free intracanal environment and preserve the survivability and function of residual host stem cells (Montero-Miralles et al., 2018).

### Cytotoxic Effects of Chemicals Used for Irrigation and Intracanal Medicaments and Their Effects on Prognosis of REPs

Optimally, the disinfecting agents used in REPs must balance between having a wide spectrum of antibacterial activity and the ability to promote attachment, proliferation, and differentiation of stem cells (Ayoub et al., 2020). The latest protocol for regenerative endodontic procedures published by the American Association of Endodontists (AAE) included irrigation by 1.5% sodium hypochlorite followed by the use of either calcium hydroxide Ca(OH)_2_ intracanal medication or low concentrations (1–5 mg/ml) of antibiotic pastes, and 17% EDTA is used in the second visit (AAE, 2018). However, these diluted antibacterial agents could still have a detrimental effect on stem cells. It was reported that, after 1 h of exposure, 1% and 5% EDTA significantly reduced the viability of stem cells of apical papilla (SCAP) and periodontal ligament stem cells, while no cell survival was observed with 1% NaOCl (Scott et al., 2018). Concentrations as low as 1 mg/ml Ca(OH)_2_ and 0.05 mg/ml TAP significantly supressed SCAP proliferation and odontogenic capacity which is coordinated with lack of continued root development and hard tissue deposition (Bi et al., 2018).

Additionally, it was found that 5 mg/ml of double antibiotic paste had significant negative effects on viability, alkaline phosphatase activity, and mineralization nodule formation of human dental pulp stem cells (DPSCs) (McIntyre et al., 2019). On another front, the current regenerative protocol could elicit indirect stem cell cytotoxicity due to failure to completely eradicate root canal pathogens which in turn activate the immune system leading to cell death (Jewett et al., 2010; Verma et al., 2017; Vishwanat et al., 2017).

### Influence of Different Irrigants and Intracanal Medicaments on the Architecture and Physical Properties of Radicular Dentin

The biomechanical performance and physical strength of immature teeth after REPs is a prerequisite for their long-term survival and success of treatment (Ali et al., 2019). Consequently, the potential added weakening of the immature roots by chemical disinfectants should be avoided. Yet, irrigants and intracanal medications used in REPs may adversely affect the mechanical and physical properties of radicular dentin. It has been reported that immature teeth treated with the regenerative disinfection protocol are more prone to fracture, especially at the cervical area, even after tooth reinforcement with adhesive restorations (Ali et al., 2019). Also, the use of EDTA, Ca(OH)_2_, and the diluted concentrations of sodium hypochlorite and antibiotic pastes currently recommended by the AAE were found to significantly decrease dentin microhardness and cause demineralization (Yassen et al., 2015). From a biological perspective, the impact of these agents on the dentinogenic capacity of stem cells should not be overlooked.

Taken together, the antibacterial efficiency of diluted irrigants/intracanal medicaments recommended by the AAE is still an open question. Moreover, eradication of root canal bacteria could be less attainable when adopting the CEC design in regenerative endodontics which cannot be overcome by increasing the concentration of the already harmful disinfectants. This entails the search for new biomimetic cell-friendly disinfecting agents that exert a broad-spectrum antibacterial action without affecting the stem cells or root dentin. This might be the key to achieving successful clinical outcomes for MIREPs.

## Alternative “Biomimetic” Strategies Could Decrease Cytotoxicity and Preserve the Integrity of Native Dental Tissues

### Alternatives to Conventional Irrigation Protocols Using Nanomaterials to Enhance the Potency and Decrease Harmful Effects

Recent research in antimicrobial strategies are aimed to enhance the potency of disinfection without having detrimental consequences. Novel disinfectants such as ozone (Kustarci et al., 2009; Silva et al., 2020b), photodynamic therapy (Zorita-García et al., 2019) and cold atmospheric plasma (Pan et al., 2013; Li et al., 2015) have been tried with variable outcomes and only limited clinical success in the field of endodontics. On the other hand, nanoscience has been a breakthrough in almost every field of science and medicine, holding special importance as regards to endodontic disinfection.

Notably, nanobubble water (NBW), has recently been introduced as a promising antimicrobial agent that could be used in many medical, dental and pharmaceutical fields. The application of NBW in Endodontics is still in its infancy, where it is recommended as a promising agent to upgrade the antimicrobial activity of root canal irrigants at lower non-cytotoxic concentrations. It exerts a kinetic force to dislodge pre-established intracanal biofilm and enhance the delivery of root canal medicaments, where it can be of beneficial in REPs. NBW was shown to be more effective in removing smear layer and at the same time preserves dentin microhardness when compared to 17% EDTA. Moreover, NBW in combination with 1.5% NaOCl was proven to be as effective as 5.25% NaOCl regarding the disinfection ability with enhanced penetration into dentinal tubules (Aksel et al., 2020; Osman et al., 2020).

Another promising biomimetic irrigant using catalytic iron-oxide nanoparticles (IO-NPs) has been introduced. The concept was to use IO-NPs in combination with hydrogen peroxide showing potent antimicrobial effects with deep penetration into the entire length of dentinal tubules. However, IO-NPs alone had modest antibacterial effects (Bukhari et al., 2018).

### Cell/Tissue Friendly Alternatives to Common Intracanal Medicaments

As previously overviewed, the use of either Ca(OH)_2_ or antibiotic combinations can cause adverse side effects during REPs. The former is responsible for root weakening even after a short period (Bukhari et al., 2018). The latter has been proven to impair functions of DPSCs even when used at the acceptable clinically recommended dosage (Diogenes et al., 2016). Recently, a nitric oxide (NO)-releasing nanomatrix gel has been introduced for controlled intracanal delivery of antibiotics (Moon et al., 2018). It was suggested that a minimal concentration of antibiotics combined with the effects of NO could be promising as an intracanal medicament, favoring root maturation and revascularization potential compared to the conventional protocol of REP (Kaushik et al., 2015; Moon et al., 2018).

Another promising alternative is the use of probiotics as a potential intracanal medicament. Local delivery of probiotics has shown promising outcomes regarding the eradication of common endodontic pathogens (Bohora and Kokate, 2017; Kim et al., 2020). Moreover, probiotics are proven to increase IL-10 release and decrease the release of IL-1 and IL-6 (Cosseau et al., 2008; Pahumunto et al., 2019) which could be beneficial in modulating the severity of apical periodontitis. However, criteria for the selection of probiotics still needs further investigation focusing on optimizing probiotics delivery to tolerate local stress and adhere to the site of application.

### Scaffolds With Dual Effect i.e., Antibacterial and Immunomodulatory Effects Could Favour Regeneration Rather Than Repair

Several recent researches have focused on the development of the smart drug delivery scaffolds to both augment root canal disinfection and reduce the risk of stem cell toxicity (Albuquerque et al., 2017; Bottino et al., 2019). An antibiotic-releasing injectable Platelet-Rich-Fibrin (I-PRF) scaffold was tested against a dual-species biofilm colonized inside the root canal, showing significant reduction in the bacterial count. The engineered biocompatible autologous scaffold that contained both antibiotics and growth factors, could favor regeneration rather than repair (Rafiee et al., 2020). Moreover, THP-1 macrophages were seeded on a concentrated growth factors (CGF) scaffold, revealing the immunoregulatory role of CGF in macrophage functional activities. The abundance of bioactive factors in the CGF extract facilitated M2 macrophage polarization and modulated cytokine secretion. These results shed light on the therapeutic potential and mechanisms of CGF in regulating the macrophage-mediated immune response, essential for tissue remodeling and healing (Luo et al., 2021).

Lately, an *in-vitro* study has evaluated a novel biomimetic scaffold composed of electrospun poly (lactic acid) nanofibers and electro-sprayed polycaprolactone with tannic acid micro-particles. This 3D cone-shaped scaffold was designed to fill the empty root canal mimicking the architecture of natural extracellular matrix (Terranova et al., 2021). Showing promising cell migration, attachment and proliferation potential, this scaffold could be further loaded with antimicrobial agents in future *in-vivo* studies.

Another interesting scaffold would be the use of decellularized tissues namely extracellular matrices (ECMs) extracted from dental pulp and dentin tissues. It was reported that these extracts have an antibacterial effect against *Streptococcus mutans*, *Streptococcus oralis* and *Enterococcus faecalis* (Smith et al., 2012). Moreover, a functional component of ECM based bio-scaffolds was recently identified, the so called, matrix-bound nanovesicles. These extracellular vesicles were found to be associated with favorable properties including enhanced angiogenic potential, stem cell recruitment and modulation of macrophage phenotype (Hussey et al., 2019; Quijano et al., 2020). Furthermore, the presence of antimicrobial peptides within injectable decellularized ECM scaffolds has been documented and might play a significant role in future studies in the field of regenerative endodontics (Jimenez-Gastelum et al., 2019; Wei et al., 2021).

### Incorporating Inflammatory Cytokines, Exosomes and Secretomes Into Scaffolds to Trigger Natural Healing/Regeneration Cascades

The parallel use of immunomodulatory and homing factors in scaffold materials has been shown to induce stem cell homing, modulate cell differentiation and indeed induce regeneration. Cathelicidin peptide (LL37) has been reported to activate the ERK signaling pathway boosting the secretion and expression of vascular endothelial growth factor (VEGF), thus promoting pulp wound healing by inducing angiogenesis (Khung et al., 2015). Likewise, interleukin (IL)-4 was incorporated in high-stiffness transglutaminase crosslinked gelatins scaffold promoting the M2 polarization and guide endogenous cell homing which represents a simple and effective strategy that can support high levels of tissue regeneration (He et al., 2019).

The use of cell-type specific exosomes (micro-vesicles) has also recently gained considerable attention in the field of regenerative medicine. These biomimetic cell products have been proven to be a driving force behind the immunomodulatory effects of mesenchymal stem cells due to their direct effect on cytokine release and M2 macrophage polarization (Zhang et al., 2014). Dental pulp-derived exosomes were shown to trigger the expression of odontogenic genes and odontogenic differentiation of Schwann cells (Huang et al., 2016; Li et al., 2021), enhance angiogenesis (Xian et al., 2018) and promote regeneration of dentin-pulp like tissues (Huang et al., 2016; Ivica et al., 2020). When incorporated within injectable hydrogels, pulp stem cell-derived exosomes were found to accelerate healing of apical periodontitis through a macrophage-dependent response (Shen et al., 2020). Another promising approach is the utilization of DPSC-conditioned medium in various regenerative endodontic applications. This secretome contains trophic factors needed to regulate the inflammatory state (Sarra et al., 2021) as well as possessing significant angiogenic, odontogenic and immunomodulatory potential (Zhou et al., 2021). Therefore, personalized utilization of various functionalized scaffolds in a case-specific manner could indeed lead to optimal regenerative outcomes through local modulation of immune response, creation of a favorable intracanal microenvironment for true regeneration and orchestrating healing cascades of periapical wounds.

## Discussion

Disinfection is a critical factor to ensure periapical healing in regenerative endodontics where literature has shown that residual infection can lead to either failure or hinder continued root development (Verma et al., 2017). Having said this, most immature permanent teeth indicated for regenerative endodontic procedures have an etiology of trauma and hence the tooth is mainly sound (Koç and Del Fabbro, 2020). Additionally, many of these teeth are subject to multiple traumatic incidents (Koç and Del Fabbro, 2020) increasing the chances of loss of these teeth. Hence a major critical objective for any type of endodontic treatment; conventional or regenerative is to ensure optimal tooth survival and retention (Elkholy et al., 2021). By minimizing tooth structure removal as we elaborated in this review, fracture resistance of such teeth can be maintained (Isufi et al., 2020; Elkholy et al., 2021), at the same time, to avoid sacrificing disinfection for the sake of preserving tooth structure, we suggest an alternative enhanced disinfection protocol that would conserve sound tooth structure while at the same time maintain an appropriate level of disinfection to allow stem cell migration and success of the final regenerative treatment.

Based on the previous review of literature, the current review suggests the possible implementation of a new concept of minimally invasive regenerative endodontic procedures (MIREPs). Starting from the access cavity design, CEC for non-decayed traumatized teeth and caries-driven-access cavity for decayed teeth, would represent more suitable tooth preserving designs, where the volume of dentin and enamel removal in case of CEC designs was found to be less than 15% which was significantly less than enamel and dentin removal in TEC (Isufi et al., 2020). Additionally, according to a recent systematic review and meta-analysis, a significant increase in fracture resistance was noted when CEC preparation was performed with preservation of tooth marginal ridges (Ballester et al., 2021). Thus this approach, accompanied by minimal mechanical preparation of root canal walls (El-Kateb et al., 2020), would offer a chance to preserve the coronal tooth structure and maximize the release of growth factors sequestered within the pericervical dentin (Tziafas et al., 2019).

As it was agreed that proper root canal disinfection through a cell- and tissue- friendly root canal irrigant and intracanal medicament is crucial for a successful outcome of minimally invasive REPs. NBW is hence recommended as an adjunct irrigant to enhance the anti-microbial activity hindered by the lesser accessibility of CEC designs. It has been proved to be efficient against multiple Gram + ve and Gram -ve bacteria with killing time ranging from 2 to 10 min (Osman et al., 2020). A suggested disinfection protocol of combined NBW with 1.5% NaOCl would represent a chemo-mechanical combination through benefiting the mechanical sheared dislodgement of attached biofilms of NBW to a shortly applied chemical disinfection of NaOCl (Aksel et al., 2020).

**FIGURE 1:**
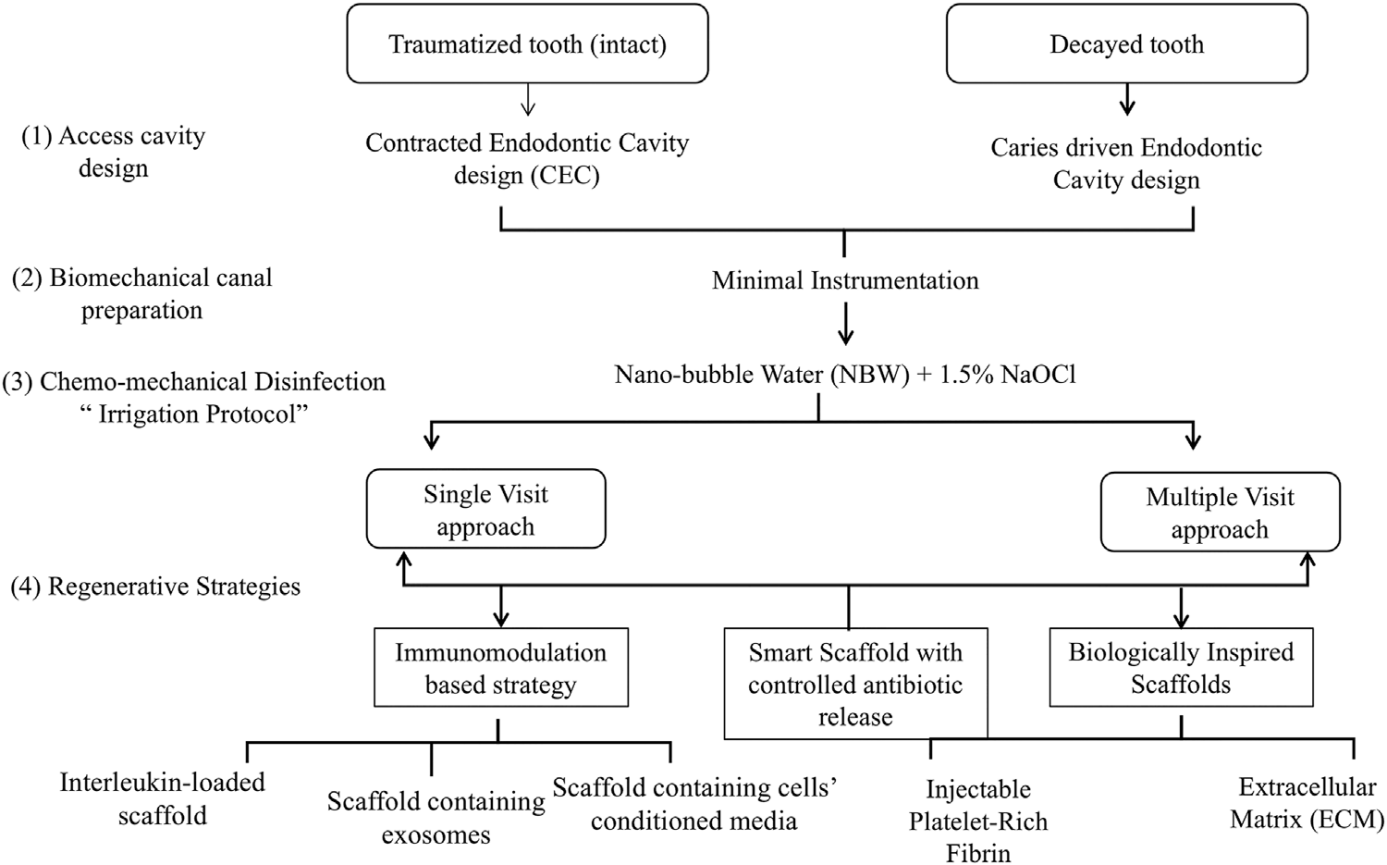
Flow Chart representing possible strategies to implement for “minimally invasive regenerative endodontic procedures (MIREPs).”

**FIGURE 2:**
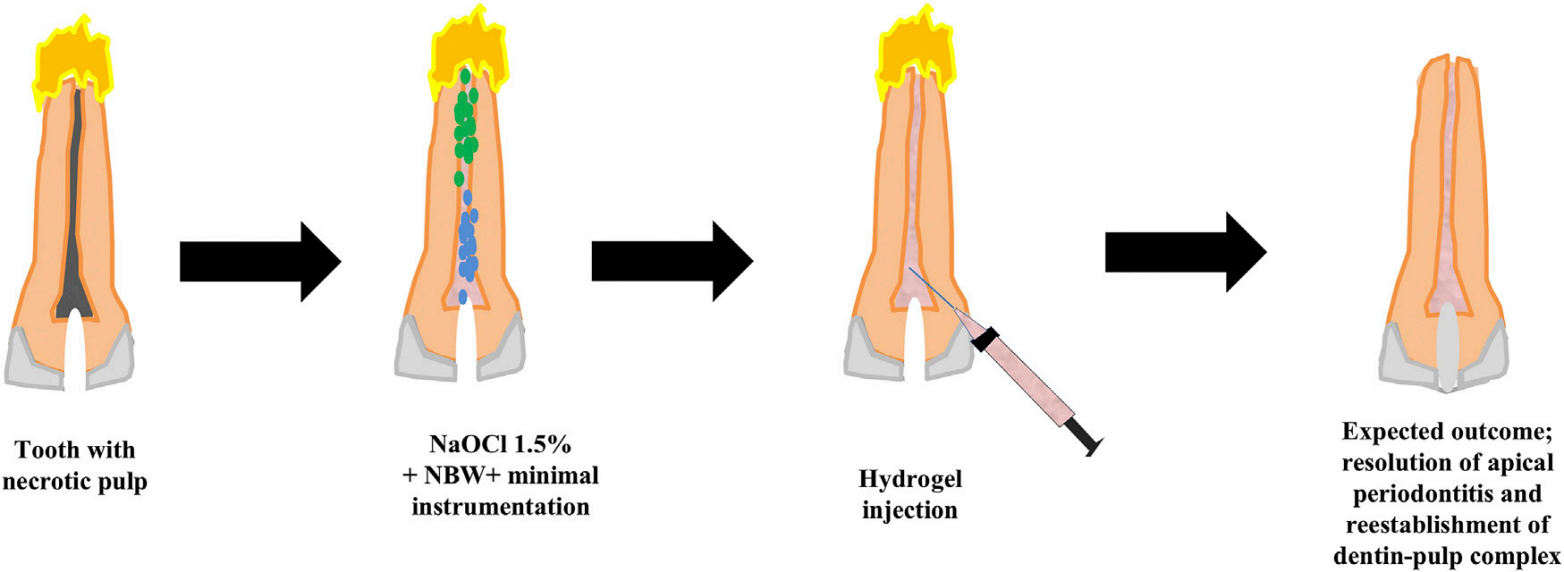
Showing the suggested clinical protocol of MIREPs. A traumatized permanent tooth with a necrotic pulp and periapical disease would be accessed using a contracted Endodontic Cavity design. This would be followed by combined irrigation of 1.5% sodium hypochlorite and nanobubble water to disinfect the canal, dislodge biofilms and release sequestered growth factors. Extracellular Matrix-based hydrogel would then be injected into the pulp space and the tooth finally sealed with bioceramic material and restored by composite resin.

Possible scenarios for implementation of a MIREP concept as well as a suggested clinical protocol are provided in Figures 1, 2 according to the clinical need of each respective case; for example whether the infection is a long-standing one or not and whether a single visit or multiple visits regimen is recommended, there can be several proposed regenerative strategies. The nature/source of the scaffold to be placed could have immunomodulatory function (Khung et al., 2015; Shen et al., 2020), antibacterial action with controlled antibiotic release (Moon et al., 2018) or a biologically inspired scaffold with dual action (Hussey et al., 2019; Rafiee et al., 2020). One suggested single visit-MIREP protocol for a traumatized permanent maxillary necrotic tooth (Figure 2) proposes a contracted endodontic cavity design followed by minimally tapered canal preparation and a disinfection regimen including combined NBW with 1.5% NaOCl followed by the placement of ECM-based hydrogel with/without cell-specific exosomes. Eventually a more optimized treatment outcome might be obtained through this novel biomimetic preservation-regeneration approach and implementing a novel concept of “minimally invasive regenerative endodontic procedures (MIREPs).”
